# An Interesting Exhibit in Medicine and Surgery at the Panama-Pacific International Exposition

**Published:** 1914-03

**Authors:** 


### Abstracts and Selections.

### An Interesting Exhibit in Medicine and Surgery at the Panama-Pacific International Exposition.

One fact alone would make the exhibit in medicine and surgery at the Panama-Pacific International Exposition the most important of any similar display at any preceding exposition, for when the world comes to San Francisco in 1915 to celebrate the completion of the Panama Canal, it will be divided in admiration of the two men who perhaps above all others are responsible, under the United States government, for the successful termination of the gigantic work. And these two men are representatives of highest honor from the science of engineering and the science of medicine: Dr. William C. Gorgas, Colonel in the United States Army Medical Corps, is the physician who undertook to preserve the lives of the canal builders in a land of malignant disease, while the toilers were operating under the guiding genius of the great Colonel George W. Goethals of the Corps of Engineers, United States Army.

Representatives of the science of medicine and surgery from every land under the sun will be present during the exposition, to pay tribute to the doctor and incidentally to study the processes whereby the ravages of a disease-ridden zone were stayed and the camp of the canal builders became the abode of health.

The element that alone would lend a distinctive character to the exhibit is the featured presentation of the methods whereby the deadly mosquito was fought in his native haunts of morass and jungle; the application of specially devised sanitary processes by which Dr. Gorgas and his men were victors in their struggle with deadly fevers, enervating malaria and others of the swarm of maladies that wait for men who penetrate those miasmic lands "where even the birds forget how to sing." A complete demonstration of these methods, as well as the equipment that, under man's uses, achieved success, will be installed for the advantage of the world by the United States government. It will excite the interest not alone of the medical fraternity, but of all such nations as are interested in the colonization of the tropics.

The Emergency Hospital, another interesting feature of the exhibit in the department of medicine and surgery, scheduled in the exposition catalog as "Group No. 35," will be a model emergency hospital, provided with its equipment entirely by exhibitors.

The law of averages works at expositions as elsewhere, and there will not be, even in 1915 in San Francisco, a suspension of the laws of gravitation, nor an annulment of the reactivities of cause and effect. Where a million people meet, there will be, in spite of all precautions to the contrary, cases of sickness, and the foolhardy will be subject to the usual percentage of disaster. Hence the necessity for an Emergency Hospital.

This Emergency Hospital will be a model, equipped by the leading manufacturers of the country with the best instruments and appliances and stocked with every drug that physicians know.

Dr. R. N. Woodward, at present in charge of the United States Marine Hospital, situated near the Golden Gate, has been appointed by the Treasury Department to assume control of the Emergency Hospital at the exposition, and he has taken great pride in assembling all of the elements, materials and equipments necessary for a model institution. How well he has succeeded, and is still succeeding, with the choice of the whole world's supply at his disposal, will be seen by the interested when the exposition is opened.

Although the entire equipment is not yet provided and while changes in what has already been selected may be made, if later proffered equipment is preferred. Dr. Woodward is sure that the Emergency Hospital at the exposition will be as near perfection as human endeavor, working in this most enlightened age, can make it.

Two superb examples of the skill of the maunfacturers of auto-ambulances will be installed, and X-ray apparatus will be placed in the X-ray ward of the hospital; sterilizing apparatus; wound-dressing appliances will be donated, and one manufacturer is providing even the spreads, with the seal of the exposition woven in the center, for the twenty beds that will be placed in the men's, women's, and isolated wards. Tables for minor and capital operations, the innumerable electric surgical appliances that human ingenuity has created, a library of medical books, a high-power microscope with photographic apparatus and dark room for the development of the negatives; and, finally, a cradle for the possible future president or countess who may insist, perhaps prematurely, on visiting the exposition.

It is not contemplated by the exposition's directorate that patients will be kept at the hospital over night, for it is to conform strictly to its classification as a hospital for emergency cases. If, however, the patient's health were to be jeopardized by removal to his home or to another hospital, he will not be removed.

The installation of the Emergency Hospital with the variety of the equipment thereof—from beds and stoves and other non-medical material to drugs, ether and operating tables and other essentially surgical or medical material—might cause a confusion in exhibits, were the scheme worked out with less careful consideration of all the exhibitors. Wherever the display normally would fall, whether in the department of Liberal Arts or Manufactures and Varied Industries, there the exhibit will be actually considered. Surgical instruments in use in the Emergency Hospital will be regarded as in the Department of Medicine and Surgery in the Palace of Liberal Arts, and will there be subjected to competitive examination with the other exhibits, although manufacturers, judging by the applications for privileges of hospital employment of products, are not unaware of the greater advantage accruing to their exhibit when shown under working conditions. In any event, the jury of awards will be careful to consider that advantage, and will not let it prejudice the displays under glass cases in the Palace of Liberal Arts.

In the meanwhile the student of municipal affairs, the expert in town policing, as well as the doctor, the surgeon and the nurse, will be vastly interested and enlightened by the model Emergency Hospital at the exposition, where any case will be cared for, from that provided by a female exercising her inalienable right to faint, or that of a child after his first lesson in the immutability of gravity's law, to that of an impulsive infant whose ambition to occupy the pretty cradle will reflect more credit on his taste than his decorum.

